# Human Rotavirus G9 and G3 as Major Cause of Diarrhea in Hospitalized Children, Spain

**DOI:** 10.3201/eid1210.060384

**Published:** 2006-10

**Authors:** Alicia Sánchez-Fauquier, Vanessa Montero, Silvia Moreno, Monica Solé, Javier Colomina, Miren Iturriza-Gomara, Ana Revilla, Isabel Wilhelmi, Jim Gray, Gegavi/VIGESS-Net Group

**Affiliations:** * Instituto de Salud Carlos III, Majadahonda, Madrid, Spain;; † Hospital La Ribera, Alzira, Valencia, Spain;; ‡ Centre for Infections Health Protection Agency, London, UK;; § Hospital Severo Ochoa, Majadahonda, Madrid, Spain

**Keywords:** Rotavirus, genotypes G, genotypes P, Spain, hospitalized, children, norovirus, astrovirus, adenovirus, research

## Abstract

In Spain, diarrhea remains a major cause of illness among infants and young children. To determine the prevalence of rotavirus genotypes and temporal and geographic differences in strain distribution, a structured surveillance study of hospitalized children <5 years of age with diarrhea was initiated in different regions of Spain during 2005. Rotavirus was detected alone in samples from 362 (55.2%) samples and as a coinfection with other viruses in 41 samples (6.3%). Enteropathogenic bacterial agents were detected in 4.9% of samples; astrovirus and norovirus RNA was detected in 3.2% and 12.0% samples, respectively; and adenovirus antigen was detected in 1.8% samples. Including mixed infections, the most predominant G type was G9 (50.6%), followed by G3 (33.0%) and G1 (20.2%). Infection with multiple rotavirus strains was detected in >11.4% of the samples studied during 2005.

## Abstract 2

A major shift in the predominant strains of rotavirus was detected.

Group A rotaviruses are a major cause of severe diarrhea in infants. In developing countries, severe diarrhea caused by human rotavirus results in an estimated 500,000 to 608,000 childhood deaths annually; worldwide, it results in ≈2 million hospitalizations (*1**,**2*).

Rotaviruses belong to the *Reoviridae* family. Viral particles are nonenveloped, and triple-layered protein capsids enclose the genome of 11 dsRNA segments. The major protein in the central layer of the viral capsid is VP6, which determines 7 different groups of rotaviruses (A–G). The outer layer of the viral capsid is composed of 2 structural proteins, VP4 (encoded by gene 4) and VP7 (encoded by gene 7, 8, or 9, depending on the strain) (*3*). These 2 proteins carry the major antigenic determinants, which elicit neutralizing antibodies and are thought to be type specific. Group A rotaviruses are widespread in humans and animals and are subdivided into distinct genotypes, G and P (*4*). Epidemiologic studies of rotavirus infections are increasingly showing that a great diversity of rotavirus strains are cocirculating in the human population throughout the world. The most common genotypes of group A rotaviruses (≈90%), which cause dehydrating gastroenteritis in infants and young children worldwide, were G1P[8], G2P[4], G3P[8], and G4P[8]; G1P[8] is the most prevalent worldwide (*5*). However, other G genotypes are epidemiologically important, such as G5 in Brazil (*6**,**7*), G9 and G10 in India (*8**,**9*), and G8 in Malawi (*10*).

In Spain, diarrhea remains an important cause of illness among infants and young children. A study conducted from 1998 through 2002 detected rotavirus in 1,155 (31%) of 3,760 specimens tested. G1 was the predominant genotype detected (53%), followed by G4 (24%), G2 (14%), G9 (6%), and G3 (2%) (*11*). The distribution of genotypes indicated a genotypic shift over time: G4 strains predominated (57%) from 1998 through 2000, whereas G1 gradually increased to account for 75% from 2000 through 2002 (*11*). Similar studies conducted in other regions of Spain indicated similar shifts in the prevalence of rotavirus genotypes (*12**,**13*).

We conducted structured surveillance among children with diarrhea who were hospitalized in 6 hospitals in Spain; our primary goals were to determine the prevalence of rotavirus diarrhea in hospitalized children, the G and P types among infecting rotavirus strains, and the temporal and geographic differences in strain distribution throughout the regions.

## Materials and Methods

### Hospitals and Patients

Stool samples were collected from children attending 6 public hospitals located in different healthcare areas throughout Spain. These hospitals intentionally represented the geographic, climatic, and ethnic diversity of Spain. Their respective catchment areas are shown in Table 1. The study was conducted between January 2005 and January 2006 and included children <5years of age who were hospitalized with acute gastroenteritis and from whom a stool sample was obtained.

**Table 1:** Description of catchment area served by each hospital in the study*

Name	Complejo Hospitalario Universitario Albacete	Complejo Hospitalario San Pedro Alcántara	Complejo Asistencial León	Hospital Fuenlabrada	Hospital Severo Ochoa	Hospital La Ribera
Municipality (province)	Albacete	Cáceres	León	Fuenlabrada (Madrid)	Leganés (Madrid)	Alzira (Valencia)
Location within Spain	Southeast	West	Northwest	Center	Center	East
Climate	Continental, semiarid	Mediterranean, continentalized	Mediterranean, continentalized	Continental	Continental	Mediterranean
No. habitants	159,518	89,029	136,414	195,133	181,248	210,637
Birth rate (per 1,000)	10.0	8.05	6.9	11.8	11.9	11.3
No. children <5 y of age	6,362	2,867	3,776	9,210	8,627	9,479

*Predominant ethnic group in each area was Caucasian.

Acute gastroenteritis was defined as >3 looser-than-normal stools within a 24-hour period or an episode of forceful vomiting and any loose stool. To enable reporting of test results to hospitals, stool specimens were labeled with the date of collection and a unique surveillance identification number. Permission for enrollment in the study was obtained from children's legal guardians, and ethical approval was obtained from the institutional review board of the Hospital de La Ribera.

### Specimen Collection and Testing

Whole stool specimens were collected and transported immediately to hospital laboratories and stored at 4°C until processing. All fecal samples were screened for enteropathogenic bacterial agents by conventional culture methods previously described (*14*).

Each month, specimens were sent to the reference laboratory (Viral Gastroenteritis Unit, National Center for Microbiology, Instituto de Salud Carlos III, Madrid, Spain). A 10% suspension in 0.1 mol/L phosphate-buffered saline (pH 7.2) was prepared and tested by reverse transcription (RT)-PCR for rotavirus, astrovirus, norovirus, and sapovirus (*11**,**15**,**16*) and by an immunochromatographic method for enteric adenoviruses (*14*).

### Nucleic Acid Extraction and G/P Rotavirus Typing

Viral RNA was extracted from 250 μL of the 10% fecal suspension by using the guanidine isothiocyanate method and the Rnaid Spin Kit (BIO 101, Anachem Bioscience, Bedfordshire, UK) according to the manufacturer's instructions, with slight modifications (*16*). RNA was eluted in 50 μL of RNase-free distilled water and stored at –20°C. To determine the G/P type patterns present in children hospitalized from 2005 through 2006, a total of 98 rotavirus strains were P typed. G and P rotavirus genotyping were performed by using RT-PCR methods as previously reported (*11**,**17*).

### DNA Sequencing and Analysis

Rotavirus amplicons were genetically characterized by nucleotide sequencing of both strands of the amplified PCR products. These products were purified by using QIAquick PCR Purification kit (Qiagen, Valencia, CA, USA) and then sequenced using an ABI PRISM BigDye Terminator Cycle Sequencing Ready Reaction Kit (Applied Biosystems, Foster City, CA, USA) on an ABI automated sequencer (Applied Biosystems, model 3700). Data analysis was performed by using Clustal for multiple alignments and neighbor-joining and maximum parsimony methods for phylogenetic analysis (Bionumerics, Kortrijk, Belgium). Spanish strains were submitted to GenBank under accession numbers DQ440613 through DQ440624.

## Results

### Microbiology

A total of 656 hospitalized children were enrolled. Enteropathogenic bacterial strains were detected in 5.0% of samples (Table 2). Astrovirus and norovirus RNA was detected in 3.2% and 12.0% samples, respectively, and adenovirus antigen in 1.8% samples.

**Table 2:** Pathogens in children <5 years of age, hospitalized with gastroenteritis, by region, Spain, 2005–2006

Pathogen	No. samples (%)						
	Albacete	Cáceres	León	Fuenlabrada	Leganés	Valencia	Total samples (%)
Rotavirus	79 (55.7)	87 (54.4)	29 (36.7)	29 (50.9)	123 (62.7)	15 (68.2)	362 (55.2)
Astrovirus	0	4 (2.5)	0	1 (1.7)	0	0	5 (0.8)
Adenovirus	3 (2.1)	0	1 (1.3)	0	7 (3.6)	0	11 (1.7)
Norovirus	8 (5.6)	18 (11.3)	5 (6.3)	3 (5.3)	9 (4.6)	0	43 (6.5)
EB*	16 (11.3)	0	4 (5.1)	3 (5.3)	9 (4.6)	0	32 (4.9)
EB + adenovirus	1 (0.7)	0	0	0	0	0	1 (0.1)
Rotavirus + astrovirus	1 (0.7)	1 (0.6)	2 (2.5)	0	5 (2.6)	1 (4.5)	10 (1.5)
Rotavirus + norovirus	2 (1.4)	7 (4.4)	3 (3.8)	2 (3.5)	16 (8.2)	0	30 (4.6)
Norovirus + astrovirus	0	0	0	2 (3.5)	3 (1.5)	0	5 (0.8)
Norovirus + astrovirus + rotavirus	0	0	0	0	1 (0.5)	0	1 (0.1)
Negative specimens	32 (22.5)	43 (26.8)	35 (44.3)	17 (29.8)	23 (11.7)	6 (27.3)	156 (23.8)
Total specimens†	142 (21.6)	160 (24.2)	79 (12.0)	57 (8.7)	196 (29.9)	22 (3.4)	656

*EB, enteropathogenic bacteria. †Percentages of isolations per total specimens in each region were 77.5, 73.1, 55.7, 70.2, 88.3, 72.7, and 76.2, respectively.

A total of 403 rotavirus strains were detected. Rotavirus was found alone in 362 (55.2%) samples but was found in another 41 samples (6.3%) as a coinfection with other viruses. The percentage of children with gastroenteritis caused by rotavirus as unique agent ranged from 36.7% in Leon to 68.2% in Valencia (Table 2).

### Rotavirus Characterization

G typing RT-PCR for rotavirus alone was performed on 362 samples positive for rotavirus but could not be determined in 10 (2.8%) samples. The G types detected, including mixed infections with multiple rotavirus strains, are shown in Table 3. Briefly, the most predominant G type was G9 (50.6%), followed by G3 (33.0%), G1 (20.2%), and G2 (7.1%); the least common G type was G4 (0.6%). G1, previously reported as the most common G type in Spain, was found in only 20.2% of rotavirus infections. With the exceptions of Valencia and Albacete, where G1 and G3, respectively, were the predominant G types, the results from all other regions showed a predominance of G9. However, even in these 2 areas, G9 was the second most common strain detected when cases with coinfection were added (26.7% and 31.6%, respectively).

**Table 3:** Rotavirus G genotypes in children <5 years of age, hospitalized with gastroenteritis, by region, Spain, 2005–2006

Rotavirus G types	No. samples (%)						Total no. samples (%) N = 352†
	Albacete, n = 79	Cáceres, n = 86	León, n = 23	Fuenlabrada, n = 28	Leganés, n = 121	Valencia, n = 15	
G1*	20 (25.3)	10 (11.6)	4 (17.4)	4 (14.3)	21 (17.4)	11 (73.3)	71 (20.2)
G2*	2 (2.5)	4 (4.7)	2 (8.7)	4 (14.3)	13 (10.7)	0	25 (7.1)
G3*	36 (45.6)	36 (41.9)	5 (21.7)	11 (39.3)	27 (22.3)	0	116 (33.0)
G4*	0	1 (1.2)	0	0	1 (0.8)	0	2 (0.6)
G9*	25 (31.6)	52 (60.5)	13 (56.5)	13 (46.4)	71 (58.7)	2 (13.3)	178 (50.6)
G1 + G2	1 (1.3)	1 (1.2)	0	2 (7.1)	0	0	4 (1.1)
G1 + G9	1 (1.3)	0	0	0	2 (1.7)	1 (6.7)	4 (1.1)
G1 + G3	0 (0.0(	0	0	0	1 (0.8)	0	1 (0.3)
G2 + G9	0	0	0	0	3 (2.5)	0	3 (0.9)
G3 + G9	2 (2.5)	16 (18.6)	1 (4.3)	2 (7.1)	6 (5.0)	1 (6.7)	28 (8.0)

*Includes mixed infections. †G typing for 10 samples could not be determined.

Common G/P combinations, infrequent patterns, and mixed-infection combinations were all detected (Table 4). G9P[8] (40%) and G3P[8] (31%) were the most common combinations detected, but G types in combination with P[6] and P[9] were also detected.

**Table 4:** G- and P-type combinations detected in 98 fully characterized strains

Genotype	No. samples	Pattern
G9 P[8]	39	Common (91%)
G3 P[8]	30	
G1 P[8]	15	
G2 P[4]	5	
G2 P[6]	1	Infrequent (3%)
G3 P[9]	1	
G9 P[6]	1	
G1+G9 P[8]	2	Mixed infections (6%)
G2+G9 P[8]	1	
G2+G9 P[4]	1	
G2+G9 and P[4]+P[8]	1	
G3+G9 and P[6]+P[8]	1	

Using DNA sequencing and phylogenetic analysis of partial sequences of the gene encoding VP7, we compared 2 G3 strains from this study with 9 G3 strains isolated previously in Spain. All G3 strains from Spain shared >99.0% homology and were more closely related to each other than to strains isolated in Italy, United Kingdom, India, and China.

## Discussion

Genetically and antigenically diverse rotavirus strains cocirculate in humans. The prevalence of rotavirus genotypes varies according to location and time. Throughout the world, genotyping and serotyping studies have identified common cocirculating rotavirus types, and G1P[8], G2P[4], G3P[8], and G4P[8] are the predominant strains. However, from time to time, other less common genotypes, such as G9P[8], G5P[8], and G8P[6], have been predominant in various countries (*5*).

In Spain, previous studies have identified G1P[8] and G4P[8] as the predominant cocirculating strains from 1996 through 2004 (*11**,**17**,**18*) (Table 5). However, in our study, conducted in 2005 and 2006, a major shift in the predominant strains was detected. G9P[8] and G3P[8] have become the predominant genotypes cocirculating in several regions of Spain, and infection with multiple rotavirus strains was detected in 11.4% of the cases studied.

**Table 5:** Predominant cocirculating rotavirus strains, Spain, 1996–2006*

Rotavirus G-type(s)	Year, %									Average (%) (n = 1,598)
	1996–1997† (n = 322)	1998–1999‡ (n = 141)	1999–2000‡ (n = 86)	2000–2001‡ (n = 200)	2001–2002‡ (n = 149)	2002–2003§ (n = 102)	2003–2004§ (n = 141)	2004-2005§ (n = 105)	2005–2006 (n = 352)	
G1 alone	68	18	27	70	79	79	79.5	50	17.1	54
G2 alone	0	1	9	23	17	16	1	11	5	9
G3 alone	2	1	12	0	0	0	17	7	24	7
G4 alone	29	68	39	3	1	0	0	26	0.6	19
G9 alone	0	11	13	3	2	3	1	5.4	39.5	9
G1+G2	0	0	0	0	1	0	1.5	0.2	1.1	0
G1+G4	1	1	0	1	0	2	0	0.4	0	1
G1+G9	0	0	0	0	0	0	0	0	1.1	0
G1+G3	0	0	0	0	0	0	0	0	0.3	0
G2+G9	0	0	0	0	0	0	0	0	0.8	0
G3+G9	0	0	0	0	0	0	0	0	7.7	1
Undet.¶	0	0	0	0	0	0	0	0	2.8	0
Total samples	322	141	86	200	149	102	141	105	362	1,608
*χ^2^ test showed annual variations in G1, G2, G3, G4, and G9 prevalence rotavirus types and in G3 + G9 mixed infections (χ^2^ = 15.50 with 8 degrees of freedom, p>0.95). †Adapted from Reference 11. ‡Adapted from Reference 17. §Adapted from Reference 18. ¶Undet., undetermined.										

Since its widespread introduction into the human population in 1995, G9P[8] has become one of the predominant viruses worldwide. In 2 separate studies conducted in Thailand (*19**,**20*), this genotype has been reported as the predominant virus circulating from 2000 through 2002 and in Brazil from 1999 through 2002 (*21*). G3P[8] has recently been reported as the predominant strain circulating in the Japanese population (*22*).

Less common G- and P-type combinations were also detected in this study. This finding may suggest either an earlier reassortment between animal and human strains, resulting in the emergence of strains such as G2P[6] and G3P[9], or zoonotic transmission to humans of an animal strain, as possibly occurred with G9P[6]. The VP4-genotypes P[6] and P[9] are reported to be associated with infection in pigs and cats, respectively. Although animal rotavirus strains replicate poorly in humans and person-to-person transmission is rare, the relatively high frequency of multiple infections detected in this study suggests that the opportunity for dual infection of a cell, and therefore reassortments, exists (*23*).

The main limitations of this study are having only 1 year of data, the minimal variations in the sampling schemes in each institution (frequency of sampling, test procedures, motivations of investigators), and the small sample size collected. Although the sampling strategy enabled monitoring for rotavirus in a large number of children, future studies with hospital-based surveillance should be initiated in different areas of Spain, and even Europe, with larger samples.

Morbidity rates worldwide and morbidity and mortality rates caused by diarrhea in developing countries remain high despite efforts to improve sanitary conditions, water quality, and the healthcare infrastructure. These high rates have driven efforts to develop a safe and effective rotavirus vaccine, and the World Health Organization has recognized that developing a vaccine is a priority for reducing infant deaths in developing countries. The level and type of protection in rotavirus disease is poorly understood, although neutralizing antibody responses are thought to be type specific. Because these responses are associated with VP7 and VP4 viral proteins, establishing the G and P genotypes of strains circulating in the human population is important. Currently, 2 candidate rotavirus vaccines are undergoing clinical trials. A multivalent vaccine directed against G1, G2, G3, G4, and P[8] and a monovalent vaccine to G1P[8] have been developed (*24**,**25*). Homotypic protection has been demonstrated for both vaccines, but the degree to which they cross-protect against less common G- and P-type combinations not included in the vaccine formulations has yet to be established, and the importance of genotype-specific protection against rotavirus disease is still under discussion (*26**,**27*). Considering that G9 rotavirus type has emerged as one of the most common rotavirus genotypes in humans around the world, and it is becoming very prevalent in some countries, future rotavirus vaccine candidates will need to provide adequate protection against disease caused by G9 viruses. Therefore, surveillance of regional networks must be maintained to document rotavirus strain distribution and prevent the appearance of new strains or new variants that could escape immune protection induced by an outdated vaccine.
